# The Importance of Risk-Reducing Prophylactic Mastectomy in Breast Cancer (BRCA) Carriers: A Case Report

**DOI:** 10.7759/cureus.5311

**Published:** 2019-08-02

**Authors:** Priyanka Ochaney, Komal Patel, Furqan Haq, Robyn Reese, Stephen Igel

**Affiliations:** 1 Osteopathic Medicine, Nova Southeastern University, Davie, USA; 2 Internal Medicine, Oak Hill Hospital, Tampa, USA; 3 Obstetrics and Gynecology, Mease Countryside, Largo, USA; 4 Obstetrics and Gynecology, Largo Medical Center, Largo, USA

**Keywords:** brca, breast cancer, mastectomy, genetic testing, dcis, hereditary breast and ovarian cancer syndrome

## Abstract

Genetic changes along with environmental exposures can play a role in the development of cancer. Individuals with significant risk factors for breast cancer including family history should be encouraged to undergo genetic testing along with breast cancer screening at an early age. Individuals who test positive for the breast cancer (BRCA) 1 or 2 gene can discuss potential risk-reducing management options, such as risk-reducing prophylactic mastectomy, with their physicians in order to protect them from the long-term consequences of developing breast cancer.

## Introduction

Mastectomy is a surgical procedure that involves the removal of one or both breasts [1]. Over 100,000 women in the United States undergo a form of mastectomy each year [1]. This procedure is performed in order to treat breast cancer as well as prevent the risk of developing breast cancer in the future [1]. In women at high-risk for breast cancer, the rate of prophylactic bilateral mastectomy increased to ~35.7% in the US from 2004-2008 [2]. Preventive mastectomy is considered appropriate in women aged 25-30 years and above [3]. According to a study by Heemskerk-Gerritsen et al., in women with a breast cancer (BRCA) mutation who underwent bilateral risk-reducing mastectomy, there was 99.6% survival [4]. Another prospective study done by Meijers-Heijboer et al. demonstrated up to 90% reduction in the incidence of breast cancer in patients who elected for bilateral prophylactic mastectomy [5]. Given the success rates of this procedure, patients at high-risk should consider prophylactic breast removal. It is uncommon to discover atypical breast tissue in mastectomy specimens; however, in our case, we discuss a unique finding of Ductal Carcinoma in Situ (DCIS) in a BRCA2 positive woman who underwent a prophylactic bilateral mastectomy.

## Case presentation

The patient is an asymptomatic 30-year-old Caucasian female with no significant past medical history who presented to the clinic for genetic evaluation. Her family history included a paternal cousin, paternal aunt, and maternal grandmother diagnosed with breast cancer. Additionally, her paternal grandfather had colorectal cancer and her maternal grandfather and uncle had prostate cancer. Given the patient’s extensive family history of malignancy, she elected for early genetic testing. She tested positive for a BRCA2 mutation c.1813dupA (p.11e605Asnfs*11), putting her at an overall increased risk for developing hereditary breast and ovarian cancer syndrome. Afterwards, her other family members underwent genetic testing, and her father and brother were found to be positive for BRCA2.

Given her status, the patient was counseled by her gynecologist to consider early childbirth to allow for the possibility of a prophylactic bilateral mastectomy and salpingo-oophorectomy in the future. After having two children, she underwent a bilateral salpingectomy. Bilateral oophorectomy was not done at that time as the patient did not want to experience early menopause. A breast magnetic resonance imaging (MRI) with and without contrast revealed Breast Imaging Reporting and Data System (BI-RADS) Category 1 with no evidence of malignancy. Although imaging studies did not show any indication of neoplasia, a breast surgeon recommended a nipple-sparing mastectomy, given her increased risk as a BRCA2 carrier.

The patient’s preoperative diagnostic mammogram showed no evidence of malignancy. She underwent bilateral nipple-sparing mastectomies with reconstruction. During a nipple-sparing mastectomy (NSM), a small incision is made to remove complete breast glandular tissue from underneath the skin and nipple [3]. Immediate reconstruction with an implant or natural breast tissue minimizes scarring and preserves the structure as well as the natural appearance of the breast [3]. Furthermore, there is a decreased risk of lymphedema when NSM is performed along with reconstruction [3].

**Figure 1:**
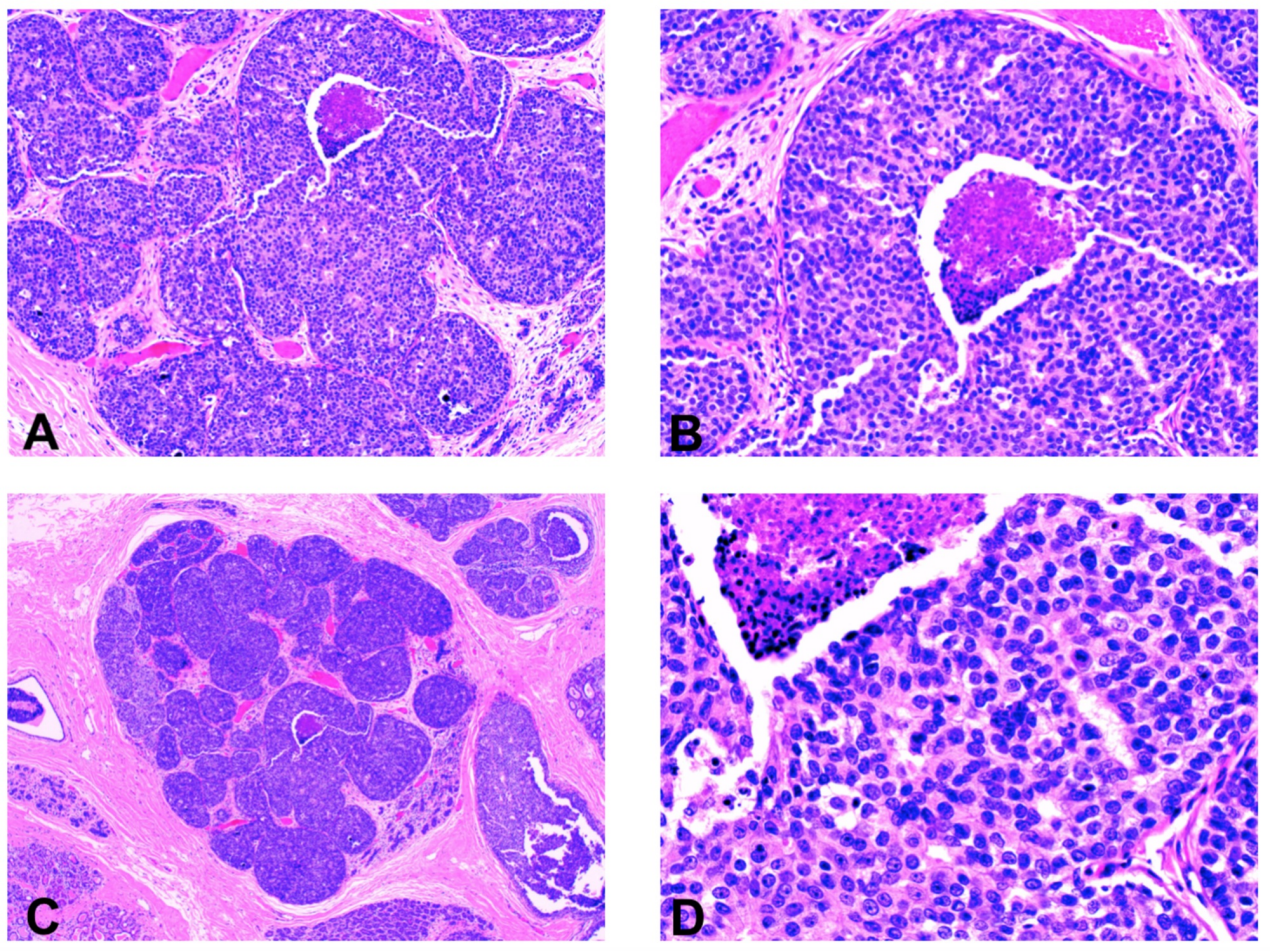
The pathology specimen demonstrates ductal carcinoma in situ (DCIS) of the left breast Figures 1A and 1B: DCIS solid type intermediate grade with comedonecrosis and calcifications. Figure 1C: DCIS with extension into lobules. Figure 1D: DCIS with margins negative for carcinoma.

Subsequent pathological report of the mastectomy specimen showed no suspicious lesions in the right breast; however, the left breast had hidden ductal carcinoma in situ (DCIS), solid type intermediate grade with comedo necrosis with calcifications [Figures 1A-1B] and extension into lobules [Figure 1C]. Margins were negative for carcinoma [Figure 1D]. No lesion was found in both nipples on pathological evaluation of the mastectomy specimen.

Following her procedure, the patient was recommended to have follow-up breast and pelvic exams, pelvic ultrasounds, and a cancer antigen-125 (CA-125) screening every 6 months. The patient is doing well and has been following up with her gynecologist for her recommended screenings biannually. She plans on undergoing a bilateral prophylactic oophorectomy in the future.

## Discussion

Breast cancer can have favorable prognostic outcomes given the patients receive proper screening and testing [6]. A gene commonly implicated in the hereditary acquisition of breast cancer is the BRCA gene [6]. BRCA1 and BRCA2 are tumor suppressor genes that help repair damaged deoxyribonucleic acid (DNA) and prevent uncontrolled cell growth [6]. Mutated BRCA1 or BRCA2 genes are not effective in repairing damaged DNA and result in unregulated cell division that can lead to cancer development at an earlier age [6]. Women with a BRCA1 or 2 mutation have a 60-85% chance of developing invasive breast cancer and a 15-65% chance of developing invasive ovarian cancer [5]. Although breast cancer screening using mammograms and MRI is optimal, further work-up including consideration for prophylactic mastectomies is essential in high-risk patients with BRCA1 or 2 mutations [6].

According to the American Cancer Society,one in eight (~12.4%) women will be diagnosed with breast cancer [7]. However, women who inherit certain genetic mutations are at an increased lifetime risk for breast cancer [7]. Although BRCA genes are commonly known to be involved in the development of hereditary breast cancer, only a small percentage of the population carries the BRCA mutation [8]. The likelihood of inheriting a BRCA1 or 2 mutation is one in 400 (~0.25%) among the general population [8]. Given the autosomal dominant pattern of inheritance, individuals with a significant family history of breast cancer should consider early genetic testing as they have a 50% chance of inheriting a mutated BRCA gene from their parents [9]. Cancers associated with BRCA1 or 2 mutations include breast, ovarian, fallopian tube, peritoneal, prostate and pancreatic cancer [10].

In individuals who test positive for BRCA1 or 2 mutations, frequent breast cancer screening at a younger age can be beneficial due to their increased lifetime risk of developing breast cancer compared to the general population [11]. According to the National Comprehensive Cancer Network (NCCN), for those at an increased risk, a mammogram and breast MRI are encouraged every year starting at age 25 to 40 years along with breast exams every 6 to 12 months starting at the age of 25 years [11]. Similarly, the American Cancer Society (ACS) suggests mammogram and breast MRI every year starting at the age of 30 years [11]. Although there is limited evidence to determine the optimal age at which to start breast cancer screening, this decision should be discussed between the patient and physician to enhance care [11]. Preemptive screening increases the likelihood of detecting malignancy at an early stage and thereby results in a better prognosis when treatment is promptly initiated [12].

Current recommendations for management of BRCA carriers include regular surveillance, chemoprevention, prophylactic mastectomy, salpingo-oophorectomy, or both [5]. While these are all feasible management options, risk-reducing prophylactic mastectomies can provide favorable outcomes in high-risk individuals [5]. A study by Scheuer et al. showed that among 29 BRCA carriers who elected for risk-reducing mastectomy, two were found to have intraductal carcinoma of the breast [12]. As illustrated in this case, the unknown diagnosis of intermediate DCIS in our patient could have progressed to invasive ductal carcinoma had she not undergone bilateral prophylactic mastectomy.

DCIS is an abnormal growth of myoepithelial cells in the mammary ducts without penetration through the basement membrane [13]. Approximately 40% of these lesions can progress to invasive disease if left untreated by invading through the basement membrane into breast stroma [13]. In order to prevent such circumstances, risk-reducing bilateral prophylactic mastectomy should be considered as this procedure has been shown to decrease the incidence of breast cancer by 90% in high-risk BRCA carriers [5].

## Conclusions

Individuals with a significant family history of breast cancer are at increased risk of developing hereditary breast and ovarian cancer syndrome. These individuals should be encouraged to undergo genetic testing along with consideration for life-saving procedures such as prophylactic mastectomy. As illustrated by this case presentation, the patient could have gone undiagnosed with DCIS if she had not undergone prophylactic mastectomy. Although prophylactic mastectomy is highly efficacious in BRCA carriers, physicians should provide education, guidance, and support to patients in order to allow them to ultimately make their own well-informed decisions.

## References

[1] (2019). Mastectomy and double mastectomy. https://www.brighamandwomens.org/surgery/surgical-oncology/resources/mastectomy.

[2] Morrow M, Mehrara B (2009). Prophylactic mastectomy and the timing of breast reconstruction. Br J Surg.

[3] Tilanus-Linthorst M, Lingsma HS, Evans D (2013). Optimal age to start preventive measures in women with BRCA1/2 mutations or high familial breast cancer risk. Int J Cancer.

[4] Heemskerk-Gerritsen BAM, Menke-Pluijmers MB, Jager A (2013). Substantial breast cancer risk reduction and potential survival benefit after bilateral mastectomy when compared with surveillance in healthy BRCA1 and BRCA2 mutation carriers: a prospective analysis. Ann Oncol.

[5] Meijers-Heijboer H, Van Geel B, Van Putten WL (2001). Breast cancer after prophylactic bilateral mastectomy in women with a BRCA1 or BRCA2 mutation. N Engl J Med.

[6] (2019). BRCA mutations: cancer risk & genetic testing. https://www.cancer.gov/about-cancer/causes-prevention/genetics/brca-fact-sheet#q1.

[7] (2019). Breast cancer facts & figures 2017-2018. American Cancer Society.

[8] Whittemore AS, Gong G, John EM (2004). Prevalence of BRCA1 mutation carriers among U.S. non-Hispanic whites. Cancer Epidemiol Biomarkers Prev.

[9] (2019). Nipple sparing mastectomy. https://www.breastcenter.com/breast-reconstruction-procedures/nipple-sparing-mastectomy/.

[10] Levine DA, Argenta PA, Yee CJ (2003). Fallopian tube and primary peritoneal carcinomas associated with BRCA mutations. J Clin Oncol.

[11] (2019). Screening, counseling, and testing guidelines. https://www.cdc.gov/cancer/breast/young_women/bringyourbrave/health_care_provider_education/guidelines.htm.

[12] Scheuer L, Kauff N, Robson M (2002). Outcome of preventive surgery and screening for breast and ovarian cancer in BRCA mutation carriers. J Clin Oncol.

[13] Cowell CF, Weigelt B, Sakr RA (2013). Progression from ductal carcinoma in situ to invasive breast cancer: revisited. Mol Oncol.

